# Alternative to General Anesthesia for a Stat Cesarean Delivery in a Patient with a Large Arteriovenous Malformation Involving the Cervicomedullary Junction in Active Labor

**DOI:** 10.1155/2020/6893587

**Published:** 2020-02-13

**Authors:** Christiano dos Santos e Santos, David A. Joyner, Cristiane A. Tuma Santos, Bernadette E. Grayson, Arthur Calimaran, Douglas R. Bacon

**Affiliations:** ^1^Department of Anesthesiology, University of Mississippi Medical Center, Jackson, MS, USA; ^2^Department of Radiology, University of Mississippi Medical Center, Jackson, MS, USA; ^3^Department of Neurobiology and Anatomical Sciences, University of Mississippi Medical Center, Jackson, MS, USA

## Abstract

A 20-year-old G1P0 patient at 38 weeks and 1 day of gestation was admitted for emergency cesarean delivery. Her past medical history was positive for cervicomedullary arteriovenous malformation (AVM) that ruptured three years before. Spontaneous vaginal delivery was contraindicated by neurosurgery. Aiming for cardiovascular stability and immediate reduction of sympathetic activity, a combined spinal epidural was successfully placed. An uneventful cesarean section was performed. The patient was transferred to the intensive care unit neurologically intact and discharged home after 8 days. This report describes an unusual anesthetic management of a patient with a large AVM in active labor.

## 1. Introduction

Cerebral arteriovenous malformations (AVMs) are rare and complex, focal, congenital, and vascular anomalies in which arterial blood flows directly into draining veins in the absence of an intervening capillary bed [1]. Normal autoregulation of the cerebral blood flow occurs through vascular resistance created by blood vessels of smaller diameter, such as arterioles and capillaries [2]. AVMs lack this crucial vascular organization leading to marked increased blood flow to the affected region. Easily recognized tortuous and dilated blood vessels are pathognomonic of AVMs [2]. In addition to these histologic aberrancies, notable cerebrovascular hemodynamic changes such as venous hypertension and reversal venous blood flow can produce rupture of the venous wall and subsequent hemorrhage [2].

The classification of AVMs is complex and based on anatomic microvascular features that are important for surgical treatment. From the anesthesia stand point, a basic anatomic classification of supra- or infratentorial is relevant since it determines the patient's positioning in the operating room. In the United States, the prevalence of AVMs in the general population is nearly 0.1% although autopsy data show that AVMs are symptomatic only in 12% of cerebral AVM cases [3]. This vascular malformation affects both sexes in approximately equal proportions, usually before the age of 40 years [4]. Hemorrhage secondary to AVM rupture is the cause of 2% of all strokes [5], and in 80–90% of the AVMs, hemorrhage is the most common initial manifestation observed [6]. The risk of the first hemorrhagic event in these patients is 2% per year, compared to 18% for those with a history of previous hemorrhage [4]. It is expected that the initial hemorrhagic event is responsible for 10%–29% of all AVM-related deaths [7].

The risk of hemorrhage from AVM rupture during pregnancy is still controversial. Conflicting opinions are found in the literature. However, the physiological changes that occur during pregnancy, especially those related to the cardiovascular system (i.e., increased blood volume and cardiac output [8]), present a strong argument that pregnancy is a robust risk factor. Based on all these factors, perioperative cardiovascular stability is mandatory and the anesthetic plan is directly correlated to the outcome. A retrospective study (1990 to 2015) with 270 North American patients concluded that there is an elevated risk of hemorrhage not only during pregnancy but also in the puerperium period [9].

This patient gave written authorization to publish this case report.

## 2. Case Description

**Figure 1:**
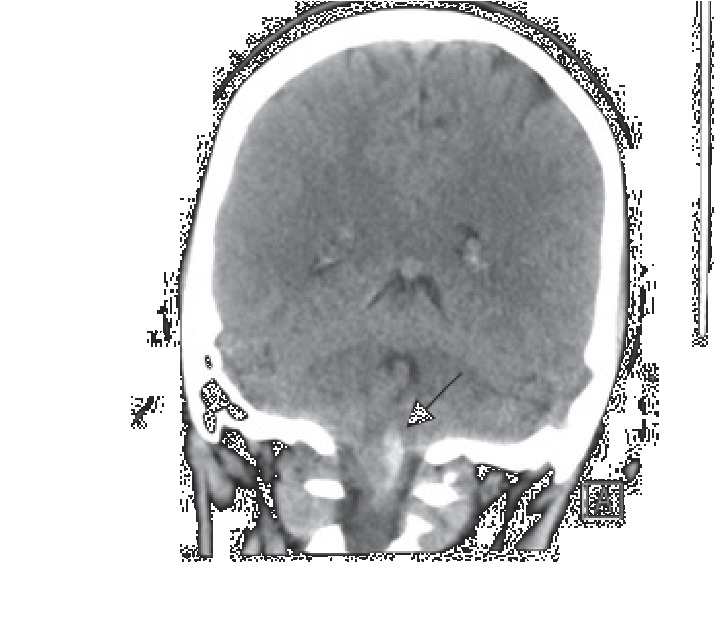
Coronal computed tomography (CT) image of the head demonstrating hyperdense intra-axial hemorrhage at the left cervicomedullary junction (arrow).

**Figure 2:**
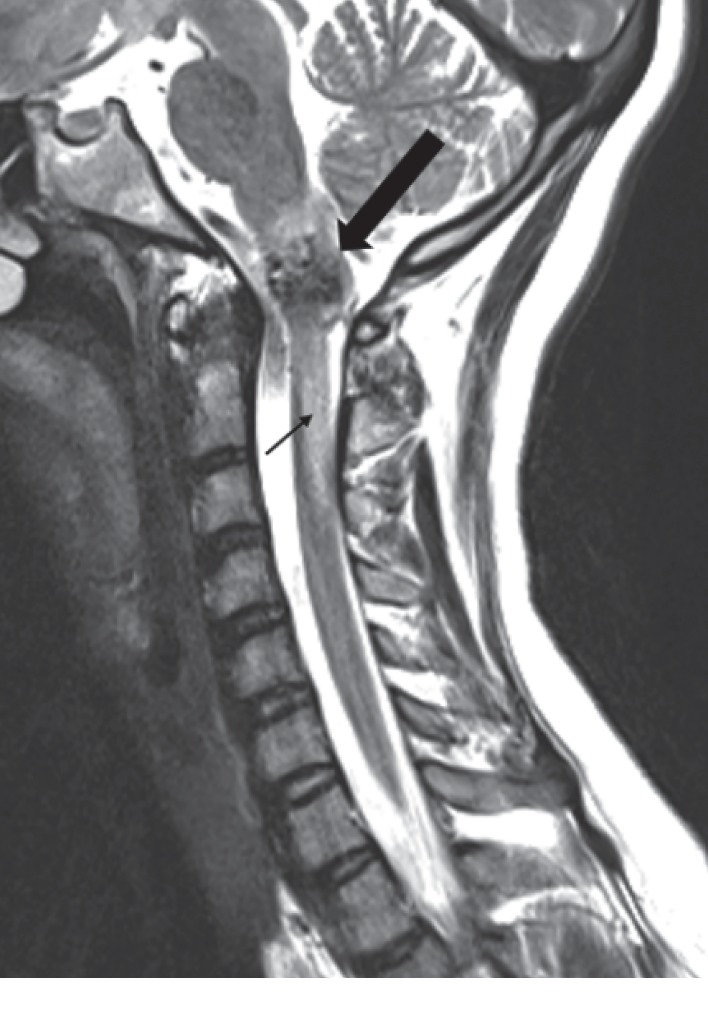
Sagittal T2-weighted MR image of the cervical spine demonstrating the expansile nidus of flow voids at the cervicomedullary junction (large arrow). Edema in the adjacent brainstem extends into the upper cervical spinal cord to the C3 level (thin arrow).

**Figure 3:**
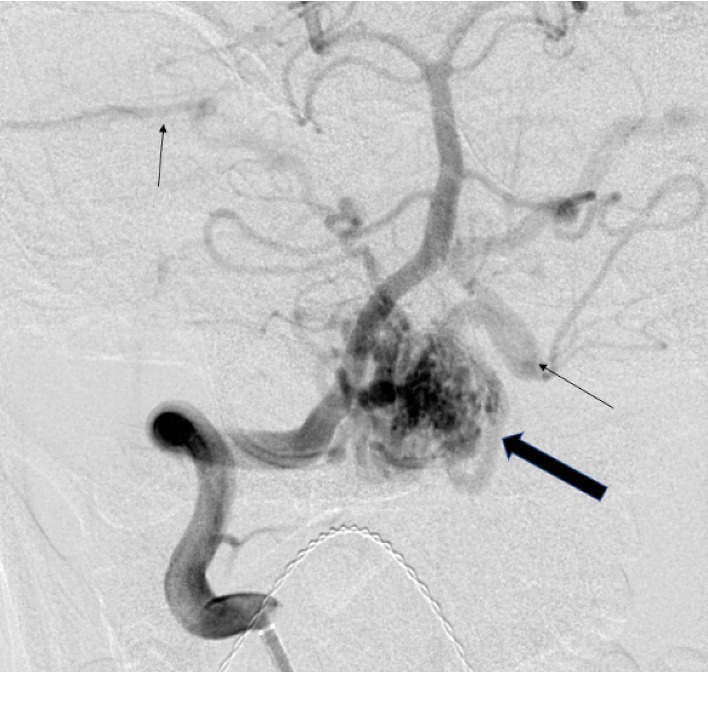
AP image from digital subtraction angiography. Right vertebral artery injection demonstrates filling of the nidus of abnormal vessels in the posterior fossa (large arrow) fed by small branches of the right vertebral artery V4 segment. Early draining veins are evident (thin arrows) which drain to the transverse sinuses.

Here, we describe a successful anesthetic management of a patient with a large AVM involving the cervicomedullary (CM) junction in active labor. A 20-year-old gravida 1, para 0 (G1P0) patient with intrauterine pregnancy at 38 weeks and 1 day of gestation was urgently admitted for primary cesarean section (CS) secondary to active labor. Prenatal care complications included maternal CM AVM. Previous neurosurgery evaluation contraindicated spontaneous vaginal delivery due to increased risk of rebleeding. Three years prior, the patient was admitted with a history of sudden onset of right-sided weakness and numbness while talking on the phone. She was unable to walk afterwards and vomited multiple times. She also complained of excruciating occipital headache and neck pain. She denied vision change, bowel/bladder incontinence, and family history of strokes or vascular malformations. The patient was awake, alert, and oriented in time, person, and place, with fluent and appropriate speech observed. Pupils were equal round and reactive to light bilaterally. V1–V3 sensation to light touch was decreased on the right side. Smile was symmetrical, and she was able to close both eyes and wrinkle forehead bilaterally. All vital signs were stable and with normal limits. In the Emergency Department, computed tomography (CT) of the head showed an acute hemorrhage within the left medulla extending into the left upper cervical spinal cord (Figure 1). MRI of the brain and cervical spine confirmed the CM AVM and demonstrated an adjacent parenchymal edema in the brainstem extending into the upper cervical spinal cord (Figure 2). CT angiogram of the head and neck showed an AVM in the right CM junction with nidus measuring approximately 1.7 cm. The patient was transferred to the neurosurgical intensive care unit (ICU). A cerebral angiogram confirmed the AVM fed by perforating branches of the right vertebral artery involving the entire CM junction, though there was more prominent involvement of the right side of the medulla and cord than the left (Figure 3). Angiography demonstrated early draining veins, draining to the bilateral transverse sinuses. Provocative testing of the AVM during the angiogram by an intra-arterial lidocaine injection showed complete loss of motor function by motor-evoked potentials (MEPs), suggestive of devastating hemiplegia if embolization occurred. After discussion of all risks and benefits, the patient and her family decided for nonsurgical intervention. She underwent clinical treatment at that time and was discharged home, neurologically intact, after 8 days.

Three years later, the patient was evaluated by her obstetrician with an intrauterine pregnancy. A neurosurgical evaluation was necessary due to her past medical history. After being evaluated, Neurosurgery suggested that vaginal delivery should be avoided due to the elevated risk of AVM rupture during labor maneuvers. At that time, CS was indicated and scheduled at 39 weeks of gestation by the Obstetrics team. The patient's prenatal care was uneventful.

One week before the scheduled CS, the patient presented to the Obstetrics Emergency Department in active labor. She was immediately transferred to the Labor and Delivery Unit. Preoperative evaluation was remarkable only for history of brainstem AVM that bled three years before the current admission. The patient and her family understood all risks of the procedure and agreed with a placement of a combined spinal continuous lumbar epidural. The procedure was uneventfully performed in the operating room after standard American Society of Anesthesiologists monitors being placed. It consisted of an intrathecal injection of 20 *µ*g of fentanyl and slow and gradual epidural injection of lidocaine 2% without epinephrine through the lumbar epidural catheter. The total amount of local anesthetic administered was 14 ml (280 mg) in 25 minutes, reaching T5 level. The CS and the postoperative period were unremarkable. At the end of the surgical procedure, 2 mg of morphine was injected in the epidural space followed by uneventful epidural catheter removal. Postoperative pain control was done with morphine patient-controlled analgesia (PCA) indicated and managed by the primary team. Tubal ligation was discussed with the patient who refused it. During that admission, Neurosurgery reevaluated the patient, but there were no new suggestions. The patient was discharged home on the postoperative day 8 without complications. She was contacted by phone one week after discharge and denied any issues at that time. During the preparation of this manuscript, the patient was contacted again and verified that she remains asymptomatic.

## 3. Discussion

Superimposed on the rarity of AVMs in general, this pathology in the brainstem is even more rare, representing approximately 5% of all encephalic AVMs [10]. Further, infratentorial lesions present a higher risk of rupture compared to supratentorial AVMs [10]. Taken together, a CM junction AVM in active labor produced a highly uncommon surgical case. The greatest dilemma we confronted in this case was the choice of an anesthetic plan that would preserve cardiovascular stability throughout active labor. Despite the fact that her vital signs were stable and with normal limits, the sympathetic response to the dilation of the uterine cervix could lead to a catastrophic event if it was not interrupted. Even though general endotracheal anesthesia, spinal, and continuous lumbar epidural would not be contraindicated in this case, our choice was to perform a combined spinal continuous lumbar epidural. The initial intrathecal injection of fentanyl provided immediate pain relief and subsequent reduction of the sympathetic activity. The patient became more comfortable, facilitating the epidural placement of the catheter. The use of lidocaine 2% without epinephrine was another quick decision that had to be made. An accidental intravascular injection of local anesthetic containing epinephrine could lead to a massive increase of cardiac output with a tragic outcome. The vasodilation secondary to the sympathetic nervous system block or sympathectomy, especially the venodilation, is imperative to accommodate the extra volume of blood following delivery and physiological autotransfusion.

Hemodynamic stability for patients with cerebral AVM is crucial. The risk of hypotension after spinal anesthesia and possible hypertension induced by vasoconstrictors is an undesirable scenario that should be avoided. On the contrary, general endotracheal anesthesia leading to sympathetic hyperactivity secondary to intubation and surgical incision could be a disaster. The combined spinal continuous epidural anesthesia offered the patient the most stable hemodynamic status, consequently decreasing the risk of hemorrhage secondary to AVM rupture, even in emergency situations as described in this case. The increased risk of AVM rupture observed in the puerperium reinforces the necessity of an efficient postoperative pain management. We encourage the utilization of combined spinal epidural technique in clinical scenarios where spontaneous vaginal delivery is contraindicated and minimal cardiovascular changes are tolerated such as in patients with intracranial vascular pathologies.

Even though brainstem AVMs during pregnancy are extremely rare, a retrospective, multicenter case series study to compare the combined anesthetic intervention with general anesthesia would be of interest. Another aspect to be compared would be cases performed electively versus stat cesarean sections. The emergent nature of the cases would definitely influence the anesthetic plan and should be compared directly for improved standard of care.

## References

[1] Ozpinar A., Mendez G., Abla A. A. (2017). Epidemiology, genetics, pathophysiology, and prognostic classifications of cerebral arteriovenous malformations. *Handbook of Clinical Neurology*.

[2] Laakso A., Hernesniemi J. (2012). Arteriovenous malformations: epidemiology and clinical presentation. *Neurosurgery Clinics of North America*.

[3] Brown R. D., Wiebers D. O., Torner J. C., O’Fallon W. M. (1996). Frequency of intracranial hemorrhage as a presenting symptom and subtype analysis: a population-based study of intracranial vascular malformations in Olmsted County, Minnesota. *Journal of Neurosurgery*.

[4] Arteriovenous Malformation Study Group (1999). Arteriovenous malformations of the brain in adults. *New England Journal of Medicine*.

[5] Gross C. R., Kase C. S., Mohr J. P., Cunningham S. C., Baker W. E. (1984). Stroke in south Alabama: incidence and diagnostic features--a population based study. *Stroke*.

[6] Kano H., Lunsford L. D., Flickinger J. C. (2012). Stereotactic radiosurgery for arteriovenous malformations, Part 1: management of Spetzler-Martin Grade I and II arteriovenous malformations. *Journal of Neurosurgery*.

[7] Wilkins R. H. (1985). Natural history of intracranial vascular malformations: a review. *Neurosurgery*.

[8] Kodogo V., Azibani F., Sliwa K. (2019). Role of pregnancy hormones and hormonal interaction on the maternal cardiovascular system: a literature review. *Clinical Research in Cardiology*.

[9] Porras J. L., Yang W., Philadelphia E. (2017). Hemorrhage risk of brain arteriovenous malformations during pregnancy and puerperium in a North American cohort. *Stroke*.

[10] Zaki Ghali M. G. (2018). Endovascular therapy for brainstem arteriovenous malformations. *World Neurosurgery*.

